# Spectral Diffusion Analysis in Patients With High Risk for Prostate Cancer: A Feasibility Study

**DOI:** 10.1002/jmri.29354

**Published:** 2024-04-05

**Authors:** Thomas A. Thiel, Birte Valentin, Tim Ullrich, Matthias Boschheidgen, Lars Schimmöller, Thomas Benkert, Rouvier Al‐Monajjed, Alexandra Ljimani, Gerald Antoch, Jonas Jasse, Eric Bechler, Hans‐Jörg Wittsack

**Affiliations:** ^1^ Department of Diagnostic and Interventional Radiology Medical Faculty and University Hospital Düsseldorf, Heinrich‐Heine‐University Düsseldorf Düsseldorf Germany; ^2^ Department of Diagnostic, Interventional Radiology and Nuclear Medicine, Marien Hospital Herne University Hospital of the Ruhr‐University Bochum Herne Germany; ^3^ MR Application Development Siemens Healthineers AG Erlangen Germany; ^4^ Department of Urology, Medical Faculty University of Düsseldorf Düsseldorf Germany; ^5^ Department of Hematology, Oncology and Clinical Oncology University Hospital Düsseldorf, Center for Integrated Oncology Aachen Bonn Cologne (CIO ABCD) Düsseldorf Germany; ^6^ Core Facility for Magnetic Resonance Imaging Medical Faculty and University Hospital Düsseldorf, Heinrich‐Heine‐University Düsseldorf Düsseldorf Germany

To reduce the incidence of overdiagnosis and enhance tumor grading of multi‐parametric‐MRI of the prostate, diffusion‐weighted imaging (DWI) has established itself as a dependable functional method for obtaining detailed information about the prostate's microstructure.^1^, ^2^ As advanced diffusion techniques evolve from the classic apparent diffusion coefficient to multi‐exponential models,^3^ these techniques have also gained more attention.^4^ One of those innovative development is the model‐free non‐negative least‐squares (NNLS) method.^5^ In a preliminary study, NNLS was successfully applied to kidney imaging, resulting in three diffusion components.

This study is a feasibility study examining the applicability of the NNLS method to the prostate as well as to differentiate between healthy and pathological tissue.

## Methods

### Study Population

The local ethics committee granted approval for the study, and all patients gave written consent to participate in the study. The patients received the in‐house standard MRI protocol according to PI‐RADS v2.1.^6^ Patients were enrolled solely if exhibiting a PI‐RADS 5 finding according to T2‐weighted imaging and DWI, indicating a high probability for presence of clinically significant cancer. In case of a PI‐RADS 5 finding an additional DWI measurement was performed prior to the application of the contrast agent. All measurement parameters can be found in Supplement 1 in the Supplemental Material.

### Model and Fitting

For analysis of the diffusion signals, we used the NNLS method with regularization to unravel the underlying diffusion components. Further information on diffusion modeling can be found in Supplement 2 in the Supplemental Material. A total of 250 diffusion components was deployed and regularized by a weighting factor μ of 0.2.

### Data Analysis

For the peripheral zone (PZ), the transition zone (TZ) as well as the PI‐RADS 5 lesion (PCA tissue) reliable regions of interest (ROI) on 3D images were applied. Hereafter mean diffusion decay signals were calculated in *Python* (v3.11.0, Python Software Foundation, Wilmington, DE, USA) for each ROI to enhance SNR and thereby stabilize NNLS analysis.^7^ NNLS analysis was performed sequentially using an in‐house developed software. More information can be found in Supplement 3 in the Supplemental Material.

## Results

### Subjects

**FIGURE 1:**
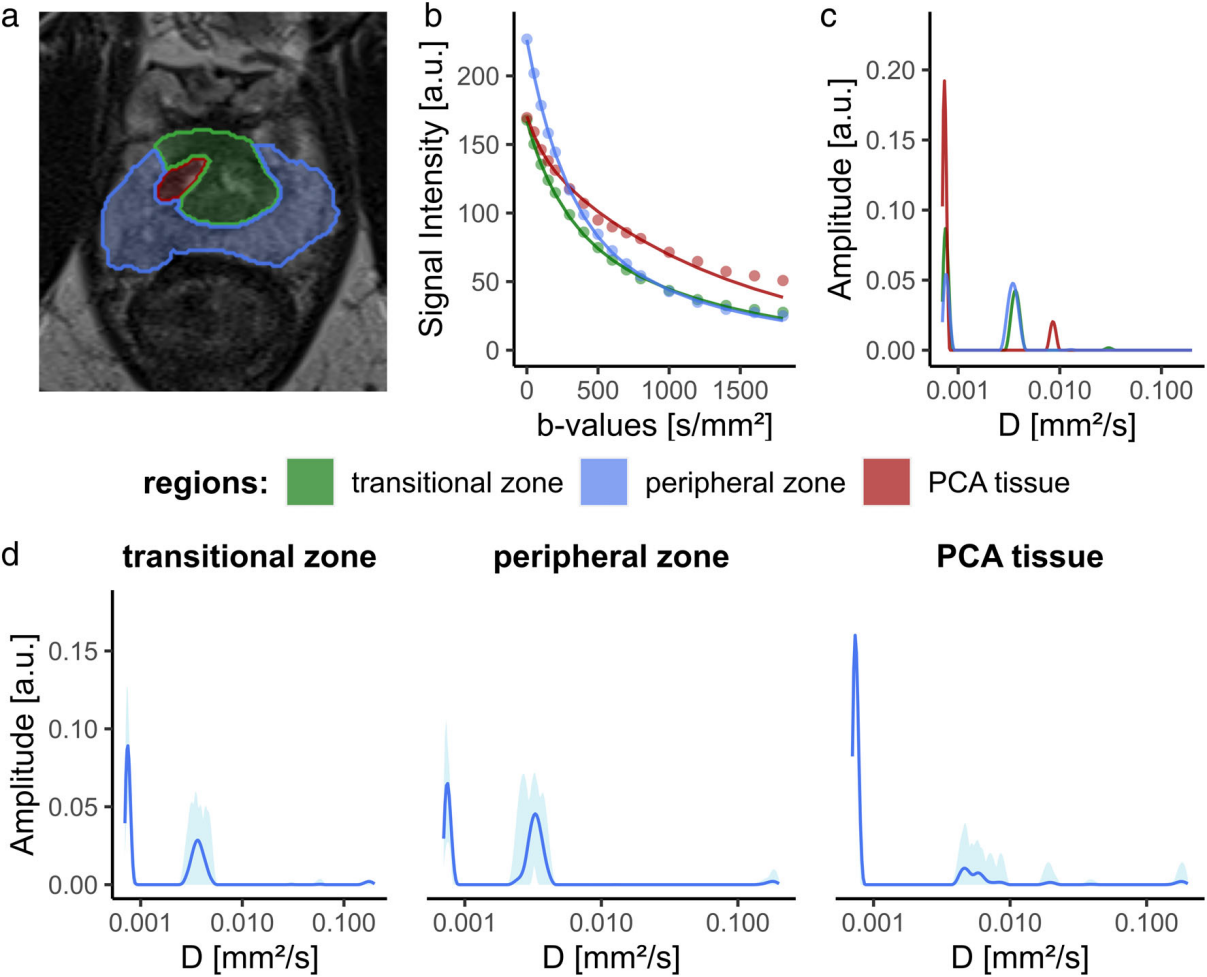
Anatomical images of the prostate gland for five different patients overlayed by interpolated regions of interest of the transitional zone, peripheral zone and PCA tissue (**a**). The mean diffusion signal curves for each region are displayed in (**b**). The resulting NNLS spectrums in (**c**) are obtained from the diffusion signals shown in (b). Mean NNLS spectra for all patients in all three analyzed regions of the prostate gland are displayed in dark blue in (**d**). The light blue background color shows minimum and maximum span for the whole group of subjects.

In total, the advanced DWI measurement was performed additively in 14 patients (57–82 years) and all data sets were of high quality. Examples of ROI placement in five different patients are shown in Fig. 1a accompanied by mean diffusion signals (b) and corresponding NNLS calculated spectra for both PZ and PCA tissue (c). For all subjects, PCA tissue exhibited lower initial signal intensities than healthy tissue and ultimately displays higher signal intensities for high *b*‐values. This is also reflected in individual spectra, which exhibit increased peak height for low D values compared to the healthy tissue for all patients.

### ROI‐Based Parameter Analysis

Both, in healthy and PCA tissue, three different diffusion components were present. Healthy and PCA tissue showed a significant difference between the three diffusion peaks (cf. Table 1). This difference is shown in Fig. 1, where both the slow as well as the intermediate diffusion components display amplitudes in the same range for the healthy tissue, while PCA tissue displays highly elevated amplitudes in the slow component. Differences in tissue compartments are further uncovered looking at the mean spectra of all subjects combined in Fig. 1d. Here, prominent differences between the TZ and PZ can be observed in the intermediate compartment, visible in a wider spread and greater variety of diffusion values present. This is manifesting in higher standard deviations for component fractions, as shown in Table 2. However, the greatest difference notable between all three tissues is the already mentioned substantially higher peak for the slow component, with a mean compartment fraction value of the PCA being around 85%, in contrast to 63% and 51% for TZ and PZ, respectively.

**TABLE 1:** *P*‐Values Comparing Different Prostate Tissues for Each Component Corrected with Bonferroni

Compartment	Tissue 1	Tissue 2	*P* Value	Significance
Fast	Transition	Peripheral	1	ns
	Transition	PC	1	ns
	Peripheral	PC	1	ns
Intermediate	Transition	Peripheral	4.35E‐04	***
	Transition	PC	1.23E‐08	****
	Peripheral	PC	6.82E‐14	****
Slow	Transition	Peripheral	2.09E‐04	***
	Transition	PC	2.93E‐09	****
	Peripheral	PC	1.13E‐14	****

*** *P* < 0.001.

**** *P* < 0.0001.

**TABLE 2:** Mean and SD Values of the Fractions for the Different Prostate Segments and the Three Detected Components

ROI	Component	Mean [%]	Std
Transition zone	Fast	1.5	0.6
	Intermediate	35.2	6.1
	Slow	63.3	6.1
Peripheral zone	Fast	1.7	1.6
	Intermediate	47.2	9.7
	Slow	51.1	8.9
Prostate cancer tissue	Fast	1.9	1.7
	Intermediate	13.7	6.4
	Slow	85.1	6.4

**FIGURE 2:**
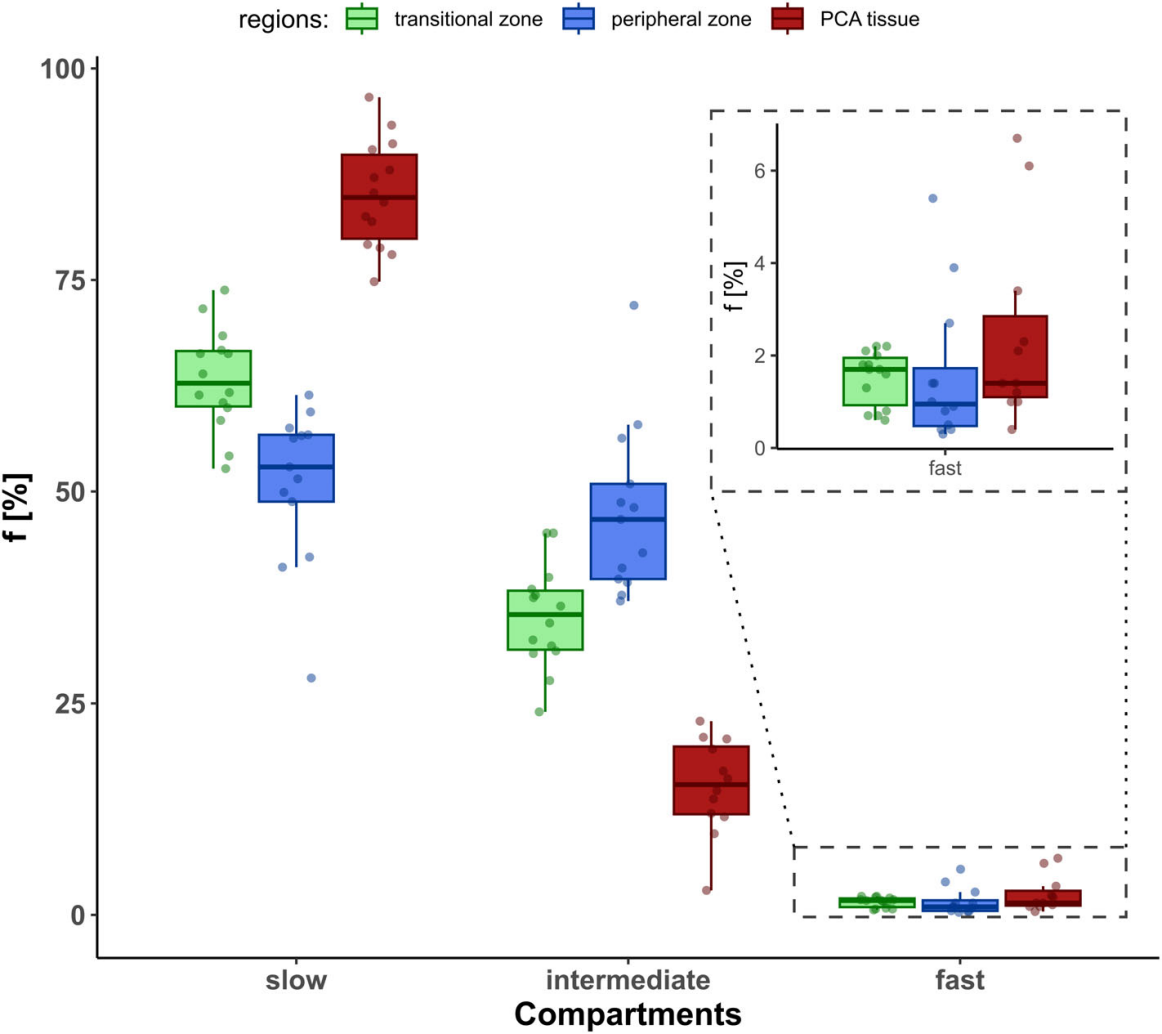
Boxplots showing distributions of subject fractions for the three compartments of the three different tissues. Zoomed in box displays boxplots for the fast component of the tissues.

Figure 2 illustrates the different fractions of each compartment in more detail. In healthy as well as PCA tissue, highly significant differences (cf. Table 1) are shown for both the slow and the intermediate compartment. Only the fast component displays no significant differences and is slightly lower than shown before.^4^

## Discussion

Our results effectively identify a spectrum of diffusion components within both normal and PCA tissue by using the unbiased model‐free NNLS method. In all three compartments, three distinct peaks were identified in the diffusion spectrum and as assigned to the fast, intermediate, and slow diffusion component, respectively. This procedure resembles those of preliminary studies of our working group in kidney tissue^8^ and studies using the IVIM model in the prostate.^4^

In contrast to previous IVIM studies,^4^, ^9^, ^10^ the NNLS approach generates diffusion spectra containing information beyond that of conventional diffusion analysis. Our study has demonstrated that the amount of individual diffusion components provides more information value than the quantitative numerical value of the individual diffusion constants. The highly significant differences between the PCA and the surrounding glandular tissue, as well as the differences in the TZ and PZ, suggests the potential for distinguishing among various types of PCA lesions. These differences are most present in the slow component, which might very well be related to compaction of the extracellular matrix in PCA tissue and loss of luminal structures.

Because of the small sample size, we could not differentiate between lesions originating from different prostate tissues, which might show different diffusion patterns. Furthermore, the absence of histopathological assessment precluded us from confirming the correlation between histopathological results and diffusion components.

For this study, we chose sequence parameters close to the original parameters used for the PI‐RADS scoring for convenience. Thereby, we were able to implement a IVIM protocol covering 16 *b*‐values with increasing averages. These parameters slightly differ from measurement parameters used in other IVIM based studies, limiting the comparability with previous studies. However, the NNLS approach showed comparable diffusion values and performed exceptionally well, distinguishing between pathologic and healthy tissue and uncovering all three components reliably by utilizing a few major advantages compared to classic IVIM. The ability of detecting a varying number of components decreases the risk of overfitting and the independence from fixed initial parameters or stopping criteria leaves room to display large changes.

## Supporting information


**Supplement 1:** In vivo MRI experiments.
**Supplement 2:** Model and fitting.
**Supplement 3:** Regions of interest and data analysis.
