# Supplementary material for: Spectral Diffusion Analysis in Patients With High Risk for Prostate Cancer: A Feasibility Study
Source: J Magn Reson Imaging. 2024 Apr 5;61(1):512–5. doi: 10.1002/jmri.29354 (PMC11645486; doi:10.1002/jmri.29354)
Supplement: Supplementary file 1 — Supplement 1: In vivo MRI experiments. Supplement 2: Model and fitting. Supplement 3: Regions of interest and data analysis. [file JMRI-61-512-s001.docx]

# Supplement 1: In vivo MRI experiments

All MRI scans were performed on a 3 Tesla whole-body MRI system (MAGNETOM Prisma, Siemens Healthineers, Erlangen, Germany) using a 60-channel body coil setup suited for prostate imaging. Basis for the anatomical and functional imaging was the PI-RADS standard MRI protocol with an added single shot EPI-DWI research application sequence which employs a tilted 2D excitation scheme to enable small FOV imaging while being robust to infolding artifacts (8) 16 b-values with 3 orthogonal directions each, using the following parameters: TE/TR = 67/3700 ms; FOV: 91x150 mm²; matrix size: 100x164; voxel size: 0.915 x 0.915 x 3 mm³; number of slices 28; fat suppression. Diffusion weighting b-values were (0, 50, 100, 150, 200, 300, 400, 500, 600, 700, 800, 1000, 1200, 1400, 1600, 1800) s/mm² with increasing averages starting with two up to 700, three up to 1400 and four averages for the last two b-values. Overall scan time was 7:14 min.

# Supplement 2: Model and Fitting

Diffusion signals are generally modelled with mono-exponential decay functions like the classic ADC analysis carried out on a variety of localities and for different pathologies. Combined with measurements of fractional anisotropy this yields an initial insight in the underlying directional diffusion processes in different tissues. For further deepening of the understanding of diffusion, intravoxel incoherent motion (IVIM) approaches have established the ability for multi-compartment analysis by implementing a superposition of multiple exponential decay terms evolving from the classic ADC approach (12,23,24). This method utilizes a fixed number of components *N* representing the expected diffusion compartments. Based on the number of measured data points *M* at b-values *b_i_* the equation (1) is fitted using algorithms such as the Levenberg-Marquardt method to determine the amplitudes *f_j_* and diffusion constants *D_j_* of each diffusion component of the IVIM analysis for the data points *y_i_*.

$$y_{i}=\sum_{i=1}^{M} f_{j}e^{-b_{i}D_{j}}, j=1, 2,\ldots,N$$

(1)

The NNLS method is based on the same idea of a superposition of exponential decay functions but takes a different approach to identify the underlying diffusion coefficients and relative amplitudes. Instead of tweaking *D_j_* and *f_j_* of a given limited number of *N*, the NNLS technique drastically increases *N* to 200 to 300 fixed diffusion coefficients along with their respective amplitudes. In addition, it enables the amplitudes to become zero, causing the corresponding decay term to vanish. Therefore, the algorithm constructs a matrix *A_ij_* containing *N* decay curves for the *M* measured b-values of each logarithmically spaced diffusion coefficient selected. Like (1) equation (2) also contains a vector denoting the relative amplitudes *s_j_* for each diffusion coefficient.

$$y_{i}=\sum_{j=1}^{M} A_{ij}s_{j}, i=1, 2,\ldots,N$$

(2)

Due to the presence of noise in MRI data, the matrix *A_ij_* is not invertible resulting in an ill-posed problem, which is resolved by the NNLS algorithm by achieving minimization of the least squares Χ² between the modelled and measured data (3).

$$X^{2}= min\left[ \sum_{i=1}^{N} \left| \sum_{j=1}^{M} A_{ij}s_{j}-y_{i} \right|^{2} \right] , with s_{j} \geq0$$

(3)

The result of this fitting is a spectrum containing sharp delta peaks representing the different compartments. To obtain a more physiological representation of the underlying diffusion within the tissue, further constraints are incorporated into equation (3). An additional regularization term is introduced to smoothen the curvature of the spectrum with a weighting factor µ resulting in (4) (25,26).

$$X^{2}= min\left[ \sum_{i=1}^{N} \left| \sum_{j=1}^{M} A_{ij}s_{j}-y_{i} \right|^{2}+ \mu\sum_{j=1}^{M} \left| s_{j+2}-2 s_{j+1}+ s_{j} \right|^{2} \right]$$

(4)

For µ = 0, the model will provide the unregularized result. Since the model returns a spectrum instead of delta peaks in this form the area under the curve is calculated for each fitted peak to calculate the relative fraction of each diffusion component.

# Supplement 3: Regions of Interest and data analysis

These ROI were achieved using the open-source imaging toolbox ITK-SNAP (v4.0, (9)). In addition to the PZ and TZ, each PI-RADS 5 lesion (hereafter referred to as PCA tissue) was also segmented in all patients resulting in whole prostate tissue coverage. All ROI were segmented by an experienced radiologist (board-certified, 5 years of experience).
For all ROI the mean fractions were calculated in R using the results from each fit.
